# Development of the designed ankyrin repeat protein (DARPin) G3 for HER2 molecular imaging

**DOI:** 10.1007/s00259-014-2940-2

**Published:** 2014-11-13

**Authors:** Robert Goldstein, Jane Sosabowski, Maria Livanos, Julius Leyton, Kim Vigor, Gaurav Bhavsar, Gabriela Nagy-Davidescu, Mohammed Rashid, Enrique Miranda, Jenny Yeung, Berend Tolner, Andreas Plückthun, Stephen Mather, Tim Meyer, Kerry Chester

**Affiliations:** 1 UCL Cancer Institute, Paul O’Gorman Building, 72 Huntley St, WC1E 6DD London, UK; 2 UCL Institute of Child Health, 30 Guilford Street, London, WC1N 1EH UK; 3 Centre for Molecular Oncology, Barts Cancer Institute, Queen Mary University of London, Charterhouse Square, London, EC1M 6BQ UK; 4 Biochemisches Institut, Universität Zürich, Winterthurerstr. 190, 8057 Zürich, Switzerland; 5 Biotherapeutics Development Unit, Cancer Research UK, Clare Hall Laboratories, Blanche Lane, South Mimms, EN6 3LD UK

**Keywords:** DARPin, HER2, Molecular imaging, Breast cancer, SPECT

## Abstract

**Purpose:**

Human epidermal growth factor receptor-2 (HER2) overexpression is a predictor of response to anti-HER2 therapy in breast and gastric cancer. Currently, HER2 status is assessed by tumour biopsy, but this may not be representative of the larger tumour mass or other metastatic sites, risking misclassification and selection of suboptimal therapy. The designed ankyrin repeat protein (DARPin) G3 binds HER2 with high affinity at an epitope that does not overlap with trastuzumab and is biologically inert. We hypothesized that radiolabelled DARPin G3 would be capable of selectively imaging HER2-positive tumours, and aimed to identify a suitable format for clinical application.

**Methods:**

G3 DARPins tagged with hexahistidine (His_6_) or with histidine glutamate (HE)_3_ and untagged G3 DARPins were manufactured using a GMP-compatible *Pichia pastoris* protocol and radiolabelled with ^125^I, or with ^111^In via DOTA linked to a C-terminal cysteine. BALB/c mice were injected with radiolabelled G3 and tissue biodistribution was evaluated by gamma counting. The lead construct ((HE)_3_-G3) was assessed in mice bearing HER2-positive human breast tumour (BT474) xenografts.

**Results:**

For both isotopes, (HE)_3_-G3 had significantly lower liver uptake than His_6_-G3 and untagged G3 counterparts in non-tumour-bearing mice, and there was no significantly different liver uptake between His_6_-G3 and untagged G3. (HE)_3_-G3 was taken forward for evaluation in mice bearing HER2-positive tumour xenografts. The results demonstrated that radioactivity from ^111^In-(HE)_3_-G3 was better maintained in tumours and cleared faster from serum than radioactivity from ^125^I-(HE)_3_-G3, achieving superior tumour-to-blood ratios (343.7 ± 161.3 vs. 22.0 ± 11.3 at 24 h, respectively). On microSPECT/CT, ^111^In-labelled and ^125^I-labelled (HE)_3_-G3 could image HER2-positive tumours at 4 h after administration, but there was less normal tissue uptake of radioactivity with ^111^In-(HE)_3_-G3. Preadministration of trastuzumab did not affect the uptake of (HE)_3_-G3 by HER2-positive tumours.

**Conclusion:**

Radiolabelled DARPin (HE)_3_-G3 is a versatile radioligand with potential to allow the acquisition of whole-body HER2 scans on the day of administration.

**Electronic supplementary material:**

The online version of this article (doi:10.1007/s00259-014-2940-2) contains supplementary material, which is available to authorized users.

## Introduction

Overexpression of human epidermal growth factor receptor-2 (HER2) enhances signal transduction through the PI3K/Akt and the Ras/Raf/MEK/MAPK pathways, enabling cancer cell proliferation and survival [1]. HER2 is overexpressed in 20 – 25 % of patients with breast cancer and gastrooesophageal cancer, and in these cancers HER2 is an established therapeutic target [2, 3]. There are a range of HER2-targeted therapies, including the monoclonal antibody trastuzumab, licensed for the treatment of breast and gastric cancers, and the trastuzumab–drug conjugate, T-DM1, licensed for the treatment of breast cancer [3, 4]. Novel therapies targeting the HER2 receptor, downstream effectors and compensatory signalling pathways are in clinical development [1].

Currently, patients are selected for anti-HER2 therapy based on histological analysis, using immunohistochemistry or fluorescence in situ hybridization (FISH) of biopsied or surgically resected tissues [5]. These analyses are limited by the use of single-site and single time-point sampling, and furthermore they fail to provide information about heterogeneity of expression or changes in expression that occur over time [6]. Dependence on histological analyses risks misclassifying patients’ HER2 status and selecting a suboptimal therapy. Discordance in HER2 status between the primary breast tumour and synchronous as well as metachronous metastases has consistently been reported [7]. HER2 status has also been reported to change, from negative to positive and vice versa, and also between metastatic disease relapses [8, 9]. Although the impact of HER2 misclassification on clinical outcomes is unclear, 20 – 30 % of patients with HER2-positive advanced breast cancer do not respond to first-line treatment combinations, despite advances in anti-HER2 therapies [10]. In the adjuvant setting, some patients with breast cancer histologically classified as HER2-negative benefit from adjuvant anti-HER2 therapy, casting doubt on their classification [11].

HER2 molecular imaging could potentially overcome the limitations of histological analysis by providing new information on HER2 expression at all metastatic sites. Radiolabelled trastuzumab has been used for this purpose, but although the pharmacokinetics of trastuzumab are well suited to therapy, its long half-life compromises tumour-to-blood ratios needed for imaging. Consequently, HER2 scans must be performed at least 2 days after radiolabelled trastuzumab administration, and even then tumours in highly vascular regions may be difficult to visualize [12]. In clinical studies, SPECT imaging with ^111^In-trastuzumab and PET imaging with both ^64^Cu-trastuzumab and ^89^Zr-trastuzumab have shown lower rates of tumour detection in patients with advanced HER2-positive breast cancer than conventional imaging, including CT [12–14]. Therefore, a novel approach is needed to overcome these limitations, potentially utilizing low molecular weight high-affinity proteins [15].

Designed ankyrin repeat proteins (DARPins) are recombinant binding proteins composed of ankyrin repeats, which stack together to make a contiguous binding surface [16, 17]. Each ankyrin repeat consists of 33 amino acids, which form a β-turn followed by two anti-parallel α-helices and a loop binding to the β-turn of the next ankyrin repeat [18, 19]. Synthetic DARPin libraries have been designed [18], from which specific binders can be selected and further evolved by methods such as ribosome display [20].

The DARPin G3 is a low molecular weight protein (14 – 15 kDa) with picomolar affinity (91 pmol/L) for HER2 [21–23]. It has a short half-life in mice (<3 min), lacks biological activity and binds to HER2 in the presence of trastuzumab and pertuzumab in vitro [21, 23]. The DARPin G3 tagged with hexahistidine (His_6_) and labelled with ^99m^Tc-tricarbonyl ([^99m^Tc(CO)_3_]^+^) can be used to visualize HER2-positive tumours [21]. Our goal was to use the DARPin G3 for routine clinical HER2 SPECT and PET imaging, and we set out to generate and evaluate different radiolabelled G3 formats to select a lead for clinical development.

The His_6_ tag can be employed for purification of recombinant proteins by immobilized metal affinity chromatography (IMAC). We evaluated the effect of the His_6_ tag on DARPin G3 biodistribution and compared it with the DARPin G3 tagged with histidine glutamate (HE)_3_, which has a negative excess charge at physiological pH. The (HE)_3_ tag has been reported to reduce background liver uptake in some cases, while still allowing tag-mediated IMAC [24, 25]. We developed a GMP-compatible *Pichia pastoris* production platform that allows cleavage of histidine-based tags after IMAC purification, enabling comparisons among variants of G3.

We hypothesized that the DARPin G3 would be capable of selectively imaging HER2-positive tumours and aimed to identify a suitable format for clinical application. Thus, we systematically investigated the effect of tag and label on the quality of imaging. First, we assessed the sensitivity and specificity of DARPin G3 radiolabelled with [^99m^Tc(CO)_3_]^+^ via a His_6_ tag in HER2-positive and HER2-negative tumour-bearing mice. Subsequently, we assessed the biodistribution of His_6_-G3, (HE)_3_-G3 and untagged G3 DARPins radiolabelled with ^111^In and ^125^I in non-tumour-bearing mice. Thus, both residualizing and non-residualizing radioisotopes were tested, as they have different cellular fates which can affect tumour-to-normal tissue ratios. Finally, the construct with the lowest normal tissue uptake was taken forward for evaluation as an imaging agent.

## Materials and methods

Details of DARPin G3 constructs (Supplementary Fig. 1), production, purification, conjugation with 1,4,7,10-tetraazacyclododecane-1,4,7-Tris-acetic acid-10-maleimidoethylacetamide (mal-DOTA) and radiolabelling are provided in the Supplementary Materials and methods.

### DARPin G3 radiolabelling

DOTA-conjugated DARPins (5 – 60 μg) in 0.2 M ammonium acetate, pH 6.5, were mixed with a solution of ^111^InCl_3_ (Covidien, The Netherlands; 10 – 30 MBq) and incubated for 2 h at 37 °C (reaction volumes 40 – 60 μl). The reactions were stopped by adding 0.1 M disodium edetate (EDTA) and the radiolabelled DARPins were purified by elution into PBS using a NAP-5 column (GE Healthcare, Little Chalfont, UK) pre-equilibrated with PBS. Radiochemical purity was determined using instant thin-layer chromatography (iTLC), using iTLC silica gel (SG) strips (Varian, Palo Alto, CA). To test for ^111^In-EDTA, iTLC strips were eluted with 0.1 M ammonium acetate containing 25 mM EDTA (final pH 5.5) in which system ^111^In-EDTA eluted to the solvent front, while ^111^In-G3 DARPin and insoluble material remained at the origin. Formation of radioactive insoluble material was evaluated using iTLC-SG eluted with 35 % ammonia (v/v)/ethanol/water (1:2:5), in which system ^111^In-DOTA-G3 DARPin and ^111^In-EDTA both had Rf values >0.5, while any insoluble material present in the reaction mixture remained at the origin. If insoluble material was detected, reaction mixtures were filtered through a 0.2-μm sterile syringe filter with a Supor membrane (Pall Life Science, Portsmouth, UK). The radiochemical purity of ^111^In-G3 DARPins was 70 – 80 % before purification and >95 % after purification (see below for specific activities, SA).

Iodine radiolabelling was performed in precoated Pierce iodination tubes (Thermo Scientific, Runcorn, UK) with unconjugated G3 DARPins (5 – 60 μg) in PBS for 10 min at room temperature, using either 10 MBq ^125^I (PerkinElmer, Llantrisant, UK) or 15 – 20 MBq ^123^I (GE Healthcare). The iodination reactions were stopped by adding sodium metabisulphite to a final concentration of 1 μM. Radioiodinated DARPins were purified by buffer exchange into PBS with a NAP-5 column. Radiochemical purity was assessed with iTLC-SG strips (Varian) using 0.1 M ammonium acetate containing 25 mM disodium EDTA (final pH 5.5) as ^123/125^I-G3 DARPin remained at the origin and free ^123/125^I eluted at the solvent front. Radiochemical purity increased from 60 – 70 % before purification to >95 % after purification (see below for SA). An equivalent molar amount of ‘cold’ potassium iodide (3.4 × 10^−10^ mol) was coadministered with ^123^I-G3 DARPin to minimize normal tissue uptake of ‘free’ ^123^I (formed by deiodination in vivo).

iTLC analysis was supplemented with size-exclusion high-performance liquid chromatography (HPLC). A Beckman System Gold 128 solvent module and a 168 UV detector module (monitoring at 280 and 220 nm; Beckman Coulter, High Wycombe, UK), combined with a Raytest GABi radiochemical detector (Raytest, Straubenhardt, Germany) were used. The radiolabelled DARPin G3 was eluted from a YMC-Pack Diol-60 (YMC Europe, Dinslaken, Germany) column, dimensions 300 mm length × 8.0 mm inner diameter, spherical shape, 5 μm particle size and 6 nm pore size, using a mobile phase of 0.2 M phosphate buffer (pH 6.8) at a flow rate of 0.5 ml/min.

### In vitro assessment

The unconjugated and DOTA-conjugated G3 counterparts had comparable subnanomolar binding affinities for the HER2 extracellular domain (ECD) assessed by surface plasmon resonance, indicating that DOTA conjugation to the C-terminus does not compromise binding to HER2 (data not shown). Saturation binding assays of radiolabelled DARPin G3 were performed with BT474 HER2-expressing human breast cancer cells (ATCC, Manassas, VA) as previously described [26]. On BT474 cell-binding assays, ^111^In-(HE)_3_-G3 and ^125^I-(HE)_3_-G3 had saturable binding and similar binding affinities to each other.

To assess stability of the label, aliquots of radiolabelled DARPin G3 were incubated in PBS or in human serum/PBS (1:1). Serum stability was assessed at 37 °C for 24 h, while PBS stability was assessed at 4, 20 and 37 °C for 24 h after radiolabelling by iTLC, as previously outlined. After incubation, the stability of radiolabelled G3 was assessed by SDS-PAGE using non-reducing conditions, followed by autoradiography using a Cyclone storage phosphor system (PerkinElmer).

### Biodistribution studies

All animal studies were ethically reviewed and performed in accordance with the UK Animals (Scientific Procedures) Act 1986, UK Home Office regulations and local regulations.

For non-tumour studies, female BALB/c mice (Charles River, Erkrath, Germany) aged 6 – 11 weeks (mean weight 19 g) received an intravenous dose of 0.3 MBq of ^111^In-G3 DARPin (2.2 μg, SA 2.0 MBq/nmol, to 4.3 μg, SA 1 MBq/nmol) or ^125^I-G3 DARPin (2.8 μg, SA 1.6 MBq/nmol, to 4.3 μg, SA 1 MBq/nmol) in 200 μl of PBS/0.1 % BSA. Mice were killed at 4 h (four mice) or 24 h (four mice) after administration. Tissues were removed and radioactivity measured in a gamma counter (1282 CompuGamma CS, LKB Wallac). Uptakes are expressed as means ± SD of the percentage of injected radioactive dose per gram of tissue (% ID/g).

For tumour studies, a single 60-day release 0.72-mg 17β-oestradiol pellet (Innovative Research of America, Sarasota, FL) was inserted into the scruff of female SCID-beige mice (Charles River) aged 6 – 8 weeks (mean weight 17 g). The following day, the mice were inoculated with BT474 cells by subcutaneous injection (7.5 × 10^6^ cells; ATCC) in PBS mixed with equal volumes of Matrigel (BD Biosciences, Oxford, UK). When tumours reached 25 – 100 mm^2^ (5 – 7 weeks after inoculation) the mice received 0.3 MBq of ^111^In-G3 DARPin (2 μg, SA 2.2 MBq/nmol) or 0.3 MBq of ^125^I-G3 DARPin (3 μg, SA 1.5 MBq/nmol) and biodistribution was calculated as outlined. Excised BT474 tumours from untreated SCID-beige mice were HER2-positive (immunohistochemistry 3+, defined as consistent with circumferential membrane staining that is complete, intense and within >10 % of tumour cells) as assessed using the HercepTest (Dako, Ely, UK) according to the manufacturer’s instructions [5].

In the trastuzumab blocking study, three female SCID-beige mice with BT474 tumours received intravenous trastuzumab (14.2 mg/kg) 24 h prior to intravenous injection of ^111^In-G3 DARPin. Three control mice received ^111^In-G3 DARPin alone. The mice were killed at 4 h after administration of 0.3 MBq ^111^In-G3 DARPin (1.9 μg per mouse, SA 2.3 MBq/nmol).

### MicroSPECT/CT imaging

Female SCID-beige mice bearing BT474 tumours were injected intravenously with either 10.5 MBq of ^123^I-G3 (2.1 MBq/μg, SA 30.1 MBq/nmol) or 8.4 MBq of ^111^In-G3 (4 MBq/μg, SA 58 MBq/nmol). Preliminary work had demonstrated that HER2-positive tumour signals on SPECT scans were compromised at lower SAs of radiolabelled G3 DARPin (data not shown). Thus, the SA for imaging was increased compared to the biodistribution studies to enhance the quality of images, but mice assessed for biodistribution and imaging studies received a similar molar dose of G3 DARPin. The radiolabelling reactions for imaging studies were performed with both a lower amount of G3 DARPin and higher radiation activity within the parameters outlined in the radiolabelling section.

Imaging was performed under 2 % isoflurane anaesthesia 4 h after administration using a microSPECT/CT animal scanner (Bioscan, Poway, CA). Helical SPECT images were acquired in 20 projections over 30 – 40 min using a four-headed camera with 4 × 9 (1.4 mm) pinhole collimators. The CT images were acquired in 180 projections with an exposure time of 1,000 ms using a peak kilovoltage of 45 kVp over 6 min. Radionuclide images were reconstructed using HiSPECT (Scivis, Göttingen, Germany) iterative reconstruction software and fused with CT images using proprietary InVivoScope (Bioscan) software.

### DARPin G3 specificity for HER2 in vivo

Mice were inoculated with tumours as previously described. G3 DARPins were radiolabelled with [^99m^Tc(CO)_3_]^+^ via a C-terminal His_6_ tag ([^99m^Tc(CO)_3_]^+^-G3 DARPin-His_6_) according to the published protocol [27]. Briefly, [^99m^Tc(CO)_3_]^+^ was prepared using an IsoLink kit (Mallinckrodt, Petten, The Netherlands) according to the manufacturer’s instructions. Following neutralization and the addition of 150 μg of G3 DARPin, the mixture was reacted at room temperature for 30 min. Radiolabelling efficiency was determined by size exclusion HPLC using the same methods as previously outlined. The product was purified from 50 % to 100 % radiochemical purity using a NAP-10 column (GE Healthcare). MicroSPECT/CT imaging was performed 1 h after administration of about 30 MBq [^99m^Tc(CO)_3_]^+^-G3 DARPin-His_6_ (10 μg, SA 44 MBq/nmol) to three mice with HER2-positive (BT474) tumours and three mice with HER2-negative (MDA-MB-468) tumours, as previously described. Mice were killed at 24 h.

### DARPin G3 specificity for HER2 in vitro

The DOTA-conjugated G3 DARPins were labelled using naturally abundant indium chloride (^nat^InCl_3_; Sigma-Aldrich, St. Louis, MO) in 0.05 M HCl solution, and unconjugated G3 DARPins were labelled using Na^nat^I (Sigma-Aldrich) in 0.1 M sodium hydroxide, using the same conditions required for ^111^In and ^125^I radiolabelling reactions, respectively.

### Competition assay

BT474 cells (ATCC) were seeded at a density of 1 × 10^6^ per well into Cellstar six-well plates (Greiner bio-one, Frickenhausen, Germany) and grown to confluence over 24 h. The confluent cells were incubated in triplicate with 0.1 nM [^99m^Tc(CO)_3_]^+^-G3 DARPin-His_6_ with or without 1,000 nM ^nat^In-DARPin or ^nat^I-DARPin in Dulbecco’s modified Eagle’s medium (DMEM) with high glucose concentration (4.5 g/L) containing 1 % fetal calf serum (Biosera, Boussens, France ) and 0.1 % sodium azide (Sigma-Aldrich) for 1 h 30 min at 20 °C. An unlabelled epidermal growth factor receptor (EGFR) binding DARPin (K. Chester, unpublished) was used as a control.

The assays were stopped by removing the medium and washing the cells with 2 ml ice-cold PBS. The cells were lysed with 1 ml of 1 M sodium hydroxide and the lysate was collected. The wells were washed twice with 1 ml of PBS. The washes were pooled with the cell lysate for analysis. The radioactivity was measured in a gamma counter. The mean percentage of radioactivity was calculated for each condition in relation to the controls treated with [^99m^Tc(CO)_3_]^+^-G3 DARPin-His_6_ alone.

### Flow cytometry analysis

BT474 (ATCC), MDA-MB-468 (ATCC) and OE-19 (Sigma-Aldrich) cells were individually prepared for flow cytometry by removal of medium and incubation with 5 ml of 0.2 % EDTA for 10 min. The cells were then transferred to tubes and centrifuged (4 °C, 4 min, 1,000 rpm). The EDTA solution was removed and 5 ml of fresh medium was added. Cells were counted and diluted to 1 million/ml, and 1 ml was used for each test condition. After washing with cold PBS, 200 μl of DARPin at 10 μg/ml was added followed by incubation for 1 h at 4 °C. Cells were washed with cold PBS and incubated with 200 μl of mouse anti-DARPin (K. Chester, unpublished) for 1 h at 4 °C. Subsequently the cells were washed with cold PBS and incubated with 200 μl of Alexa Fluor® 488 goat anti-mouse IgG (Life Technologies, Paisley, UK) for 30 min at 4 °C. After further washing with cold PBS, the cells were suspended in 500 μl of cold PBS. Samples were analysed on a CyAn ADP high-performance flow cytometer (Becton Dickinson, Oxford, UK); cells were gated according to size scattering, forward scattering and pulse width so only single cells were analysed. A total of 10,000 cell events were recorded per sample and data were analysed using FlowJo software (Tree Star, Ashland, OR).

### Statistical analysis

An independent samples *t* test was performed with SPSS Statistics 21 software (IBM, Armonk, NY) to compare normal liver uptake between the different radiolabelled G3 DARPins (*p* values <0.05 were considered statistically significant).

## Results

### In vitro stability of label

Over 24 h, ^111^In-(HE)_3_-G3 DARPin was stable in PBS at 4, 20 and 37 °C and in serum at 37 °C, as >95 % of radiation activity remained bound to (HE)_3_-G3 and each sample contained a single-sized radiolabelled protein that had the appropriate molecular weight (Supplementary Table 1 and Supplementary Fig. 2).

### Specificity for HER2

**Fig. 1:**
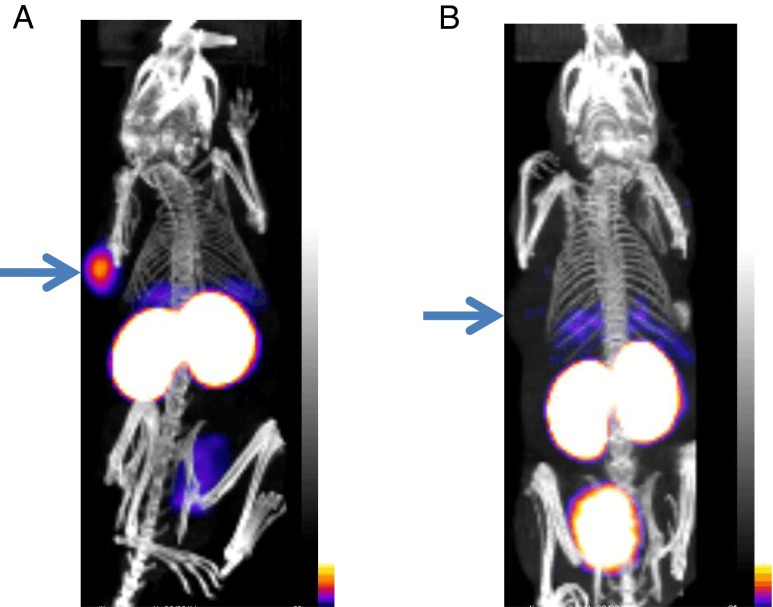
microSPECT/CT scan of [^99m^Tc(CO)_3_]^+^-G3 DARPin-His_6_ at 1 h in SCID-beige mice bearing: **a** HER2-positive human breast tumour (BT474), and **b** HER2-negative human breast tumour (MDA-MB-468). Tumours (*arrows*) assessed at same sensitivity level

HER2 tumour specificity was first established with [^99m^Tc(CO)_3_]^+^-G3 DARPin-His_6_, which had 3.5-fold higher uptake in HER2-positive (BT474) tumours than in HER2-negative (MDA-MB-468) tumours (3.5 ± 1.1 vs. 1.0 ± 0.2 % ID/g at 24 h after administration). Uptake in normal tissues was similar in the HER2-positive and HER2-negative tumour-bearing mice (Table 1). The differences in tumour uptake were also apparent on microSPECT/CT scanning (Fig. 1). However, [^99m^Tc(CO)_3_]^+^-G3 DARPin-His_6_ did not appear optimal for imaging, since the tumour-to-liver ratios were low (1.2:1 at 24 h) and the tumour-to-blood ratios were not optimal (26:1 at 24 h). Thus, there was a need to optimize G3 by assessing the effects of histidine-based tags on the biodistribution of G3 in normal tissue.

**Table 1:** Biodistribution of [^99m^Tc(CO)_3_]^+^-G3 DARPin-His_6_ in HER2-positive and HER2-negative tumour-bearing SCID-beige mice

Organ	HER2-positive (BT474) tumours at 24 h, mean % ID/g ± SD (*n* = 3)	HER2-negative (MDA-MB-468) tumours at 24 h, mean % ID/g ± SD (*n* = 3)
Tumour	3.5 ± 1.1	1.0 ± 0.2
Spleen	0.9 ± 0.2	1.0 ± 0.3
Kidney	108.0 ± 7.7	108.6 ± 10.6
Liver	3.0 ± 0.2	2.7 ± 0.7
Lung	0.5 ± 0.1	0.5 ± 0.2
Blood	0.1 ± 0.0	0.1 ± 0.00
Muscle	0.3 ± 0.1	0.2 ± 0.1

**Fig. 2:**
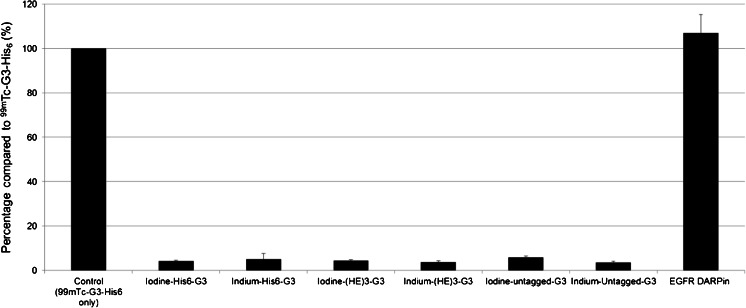
Competition assay using BT474 cells, treated with 0.1 nM [^99m^Tc(CO)_3_]^+^-G3 DARPin-His_6_ with or without 1,000 nM cold DARPins. Each condition was assessed in triplicate and expressed as percentage of radioactivity in relation to the control treated with 0.1 nM [^99m^Tc(CO)_3_]^+^-G3 DARPin-His_6_ alone (means ± SD)

A competition assay showed that the His_6_-G3 labelled with cold indium and cold iodine, (HE)_3_-G3 and untagged G3 all competed with [^99m^Tc(CO)_3_]^+^-G3 DARPin-His_6_ for binding to HER2-positive human breast cancer cells (BT474). By contrast, non-labelled EGFR-targeting DARPin did not compete with [^99m^Tc(CO)_3_]^+^-G3 DARPin-His_6_ for binding to BT474 cells (Fig. 2).

**Fig. 3:**
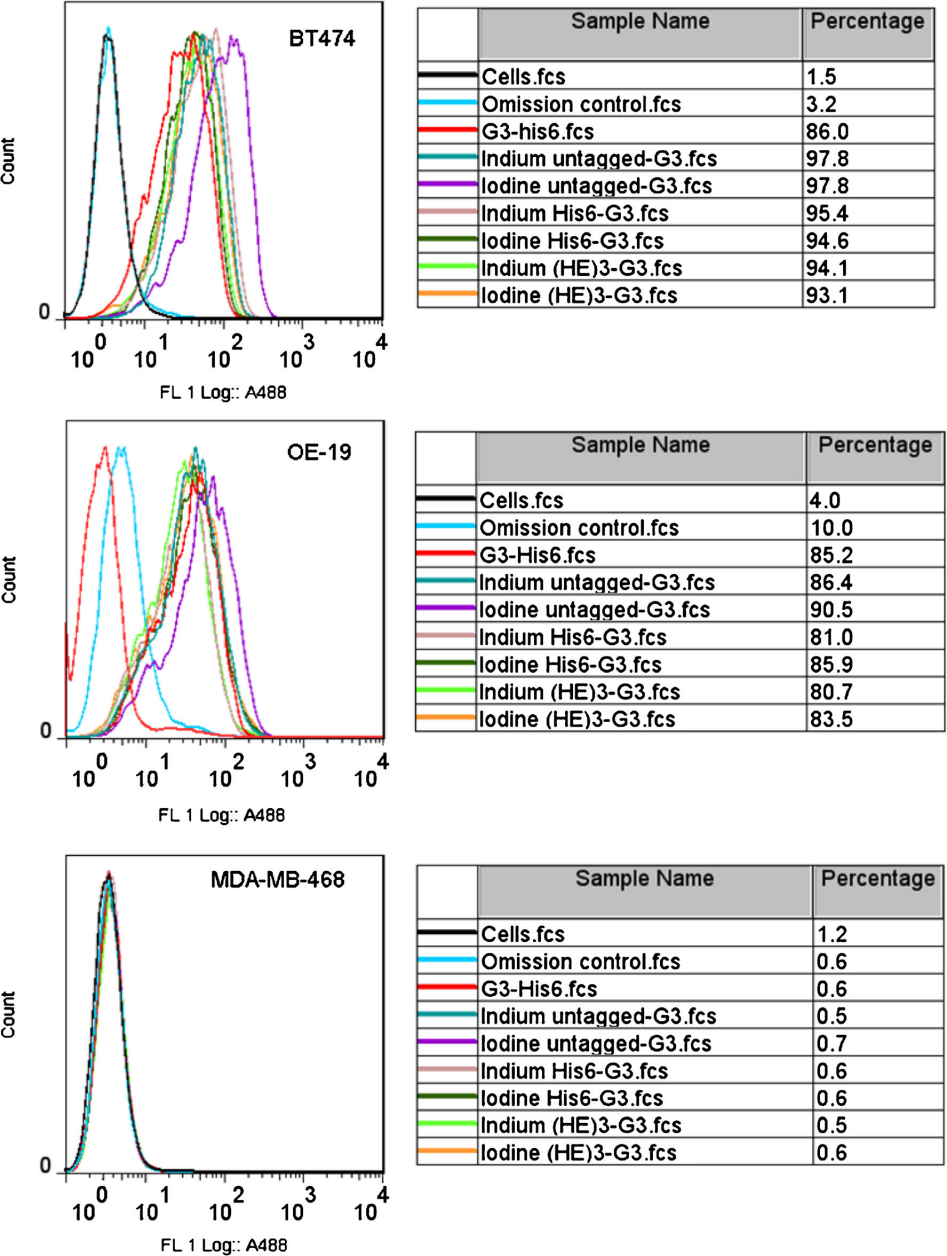
Flow cytometry of G3 DARPins labelled with cold indium and cold iodine assessed in human HER2-positive breast cancer cells (BT474) and gastrooesophageal junction (OE-19) adenocarcinoma cells, as well as in human HER2-negative breast adenocarcinoma cells (MDA-MB-468). G3-His_6_ was unlabelled and omission controls were treated with cells and antibodies in the absence of G3 DARPin. Percentages are percentage of cells with G3 DARPin binding (*fcs* flow cytometry study)

Flow cytometry demonstrated that G3 DARPins labelled with cold indium and cold iodine bound to HER2-positive human breast cancer cells (BT474) and HER2-positive human gastrooesophageal junction (OE-19) adenocarcinoma cells, but did not bind to HER2-negative human breast adenocarcinoma cells (MDA-MB-468). The non-labelled G3 DARPin-His_6_ and the assessed cold-labelled DARPins demonstrated HER2 specificity in vitro (Fig. 3). Thus, HER2 specificity of His_6_-G3, (HE)_3_-G3 and untagged G3 DARPins in vitro was confirmed.

### Biodistribution in non-tumour-bearing mice

**Fig. 4:**
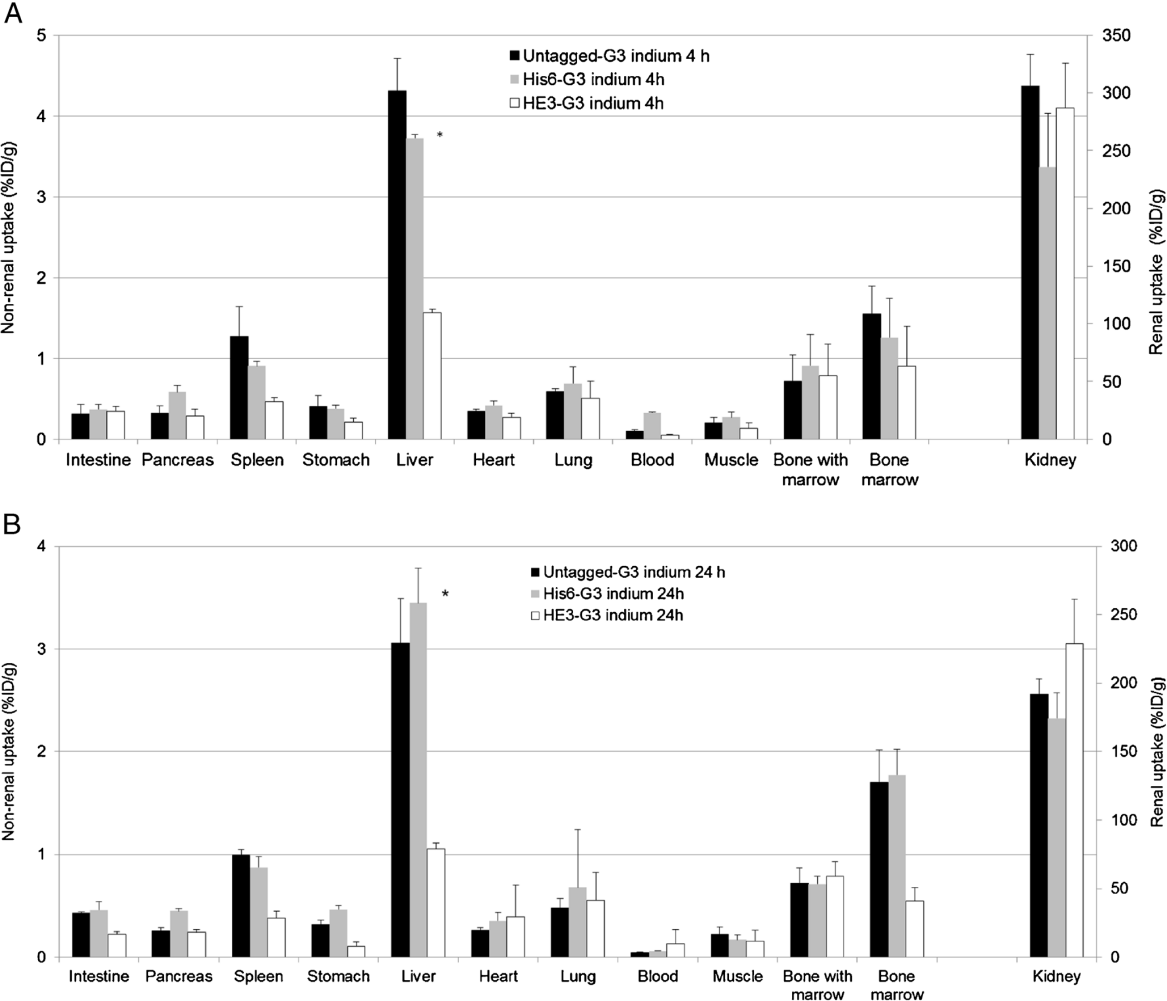
Biodistribution of ^111^In-G3 DARPins **a** 4 h and **b** 24 h after administration in female BALB/c mice (mean % ID/g ± SD). *p < 0.05, DARPins (HE)_3_-G3 vs. His_6_-G3 and untagged G3 (liver uptake)

Normal tissue uptake of His_6_-G3, (HE)_3_-G3 and untagged G3 DARPins radiolabelled with ^111^In and ^125^I was first assessed in non-tumour-bearing mice. ^111^In-(HE)_3_-G3 DARPin had lower uptake in the spleen, stomach, liver and bone marrow at 4 h and 24 h than ^111^In-His_6_-G3 and ^111^In-untagged-G3. In other normal tissues, ^111^In-(HE)_3_-G3 had either similar or lower uptake at 4 h and 24 h than its ^111^In radiolabelled counterparts (Fig. 4 and Supplementary Table 2).

At 4 h, ^111^In-(HE)_3_-G3 had significantly lower liver uptake than ^111^In-His_6_-G3 (*p =* 0.001) and ^111^In-untagged-G3 (*p =* 0.001). Also at 24 h after administration, ^111^In-(HE)_3_-G3 liver uptake was significantly lower than that of ^111^In-untagged-G3 (*p =* 0.002) and ^111^In-His_6_-G3 (*p =* 0.001). Interestingly, there was no significant difference in liver uptake between the ^111^In-untagged-G3 and ^111^In-His_6_-G3 at 4 and 24 h after administration (Fig. 4). This suggests a specific favourable influence of the (HE)_3_-tag, rather than some level of liver targeting of the His_6_ tag. Bone uptake was largely attributed to the marrow, as marrow uptake was similar to intact bone uptake. Kidney uptake was greater than 200 % ID/g for all ^111^In-G3 DARPins at 4 h (Fig. 4 and Supplementary Table 2).

**Fig. 5:**
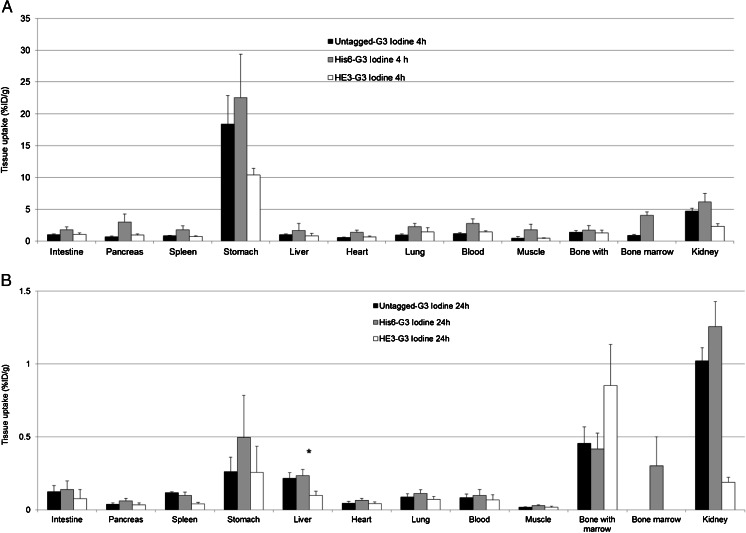
Biodistribution of ^125^I-G3 DARPins **a** 4 h and **b** 24 h after administration in female BALB/c mice (mean % ID/g ± SD). *p < 0.05, DARPins (HE)_3_-G3 vs. His_6_-G3 and untagged G3 (liver uptake)

In the study of the radioiodinated DARPins (Fig. 5), where we also have to take enzymatic dehalogenation into account, ^125^I-(HE)_3_-G3 and ^125^I-untagged-G3 showed the lowest uptake in all normal tissues at 4 h. At 24 h, liver uptake of ^125^I-(HE)_3_-G3 was significantly lower than those of ^125^I-untagged-G3 (*p* = 0.004) and His_6_-G3 (*p =* 0.003). There was no significant difference in normal liver uptake between ^125^I-His_6_-G3 and ^125^I-untagged-G3 at 4 h and 24 h (Fig. 5 and Supplementary Table 3).

Normal tissue uptake of ^125^I was <1.3 % ID/g for the ^125^I-labelled G3 DARPins at 24 h, while for ^111^In-G3 DARPins normal tissue uptake was better maintained between 4 and 24 h (Figs. 4 and 5).

### Biodistribution in mice bearing HER2-positive tumours

**Fig. 6:**
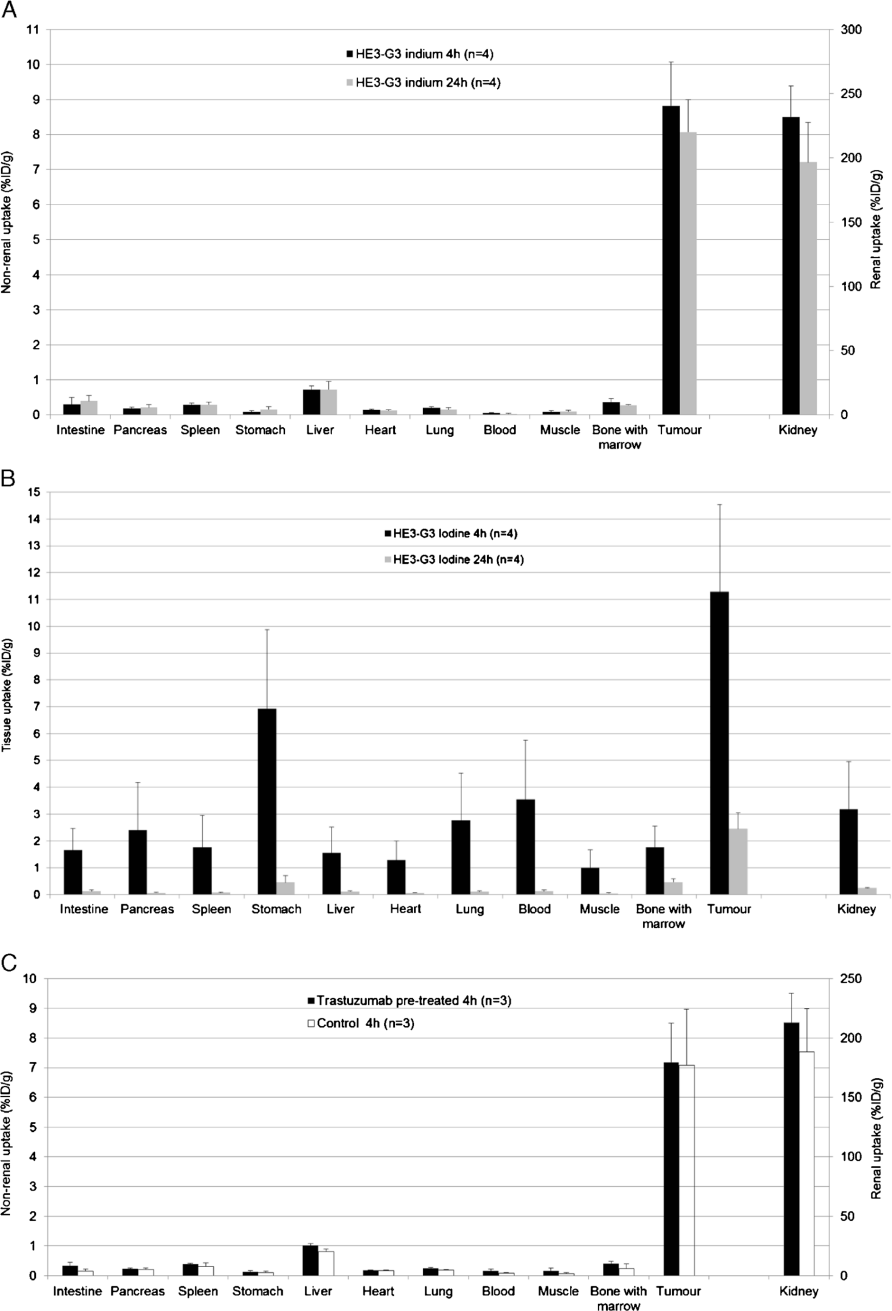
Biodistribution of (HE)_3_-G3 in female SCID-beige mice bearing HER2-positive human breast tumours (BT474) (mean % ID/g ± SD) pretreated with trastuzumab 24 h prior to DARPin administration (**a** ^111^In-(HE)_3_-G3 DARPin, **b** ^125^I-(HE)_3_-G3 DARPin. **c** ^111^In-(HE)_3_-G3 DARPin). Controls received only ^111^In-(HE)_3_-G3

The biodistribution of (HE)_3_-G3 was assessed in HER2 tumour-bearing mice, and this construct was chosen on the basis of its lower normal tissue uptake (Figs. 4 and 5), which could facilitate imaging of HER2-positive tumours by achieving greater contrast between tumours and normal tissues. The normal tissue uptake of both ^111^In-(HE)_3_-G3 and ^125^I-(HE)_3_-G3 in tumour-bearing mice was similar to that in non-tumour-bearing mice (Supplementary Figs. 3 and 4). Similarly, normal tissue uptake of ^111^In-(HE)_3_-G3 in tumour-bearing mice was lower than that of ^125^I-(HE)_3_-G3 at 4 h, except in the kidneys. At 24 h, the differences in normal tissue uptake between ^111^In-(HE)_3_-G3 and ^125^I-(HE)_3_-G3 were smaller (Supplementary Table 4). Kidney uptake of ^111^In-(HE)_3_-G3 was higher than that in other tissues tested; it peaked at 4 h after administration at 232.0 ± 24.1 % ID/g and decreased to 196.5 ± 31.0 % ID/g at 24 h after administration (Fig. 6a, b and Supplementary Table 4).

The measured tumour uptake of ^125^I-(HE)_3_-G3 and ^111^In-(HE)_3_-G3 was similar at 4 h (11.3 ± 3.2 and 8.8 ± 1.3 % ID/g, respectively). However, the radioactivity from ^111^In-(HE)_3_-G3 in the tumour was better maintained, so that by 24 h ^111^In-(HE)_3_-G3 tumour radioactivity was over threefold higher than that of ^125^I-(HE)_3_-G3 (8.1 ± 0.9 vs. 2.5 ± 0.6 % ID/g; Fig. 6a, b and Supplementary Table 4).

In comparison with ^125^I-(HE)_3_-G3, ^111^In-(HE)_3_-G3 had higher tumour-to-normal ratios for all tissues except the kidneys at 4 h (Table 2). Tumour-to-blood ratios for ^111^In-(HE)_3_-G3 were 174.7 ± 26.1 at 4 h and 343.7 ± 161.3 at 24 h, compared with 4.4 ± 3.4 at 4 h and 22.0 ± 9.6 at 24 h for ^125^I-(HE)_3_-G3 (Table 2).

**Table 2:** Tumour-to-normal tissue ratios of ^111^In-(HE)_3_-G3 and ^125^I-(HE)_3_-G3 in female SCID-beige mice bearing HER2-positive human breast tumours (BT474)

Tissue	Tumour-to-tissue ratio			
	^111^In-(HE)_3_-G3		^125^I-(HE)_3_-G3	
	4 h (*n* = 4)	24 h (*n* = 4)	4 h (*n* = 4)	24 h (*n* = 4)
Tumour	1.0 ± 0	1.0 ± 0	1.0 ± 0	1.0 ± 0
Intestine	39.4 ± 21.1	21.8 ± 5.1	8.0 ± 3.6	20.8 ± 10.9
Pancreas	50.1 ± 15.3	41.5 ± 12.7	6.6 ± 4.2	36.2 ± 11.6
Spleen	32.3 ± 9.6	28.6 ± 5.0	8.5 ± 5.1	34.2 ± 11.2
Stomach	148.8 ± 91.1	67.5 ± 46.6	1.3 ± 0.8	6.4 ± 2.501
Kidney	0.04 ± 0.008	0.04 ± 0.005	4.3 ± 2.2	9.8 ± 2.5
Liver	12.4 ± 1.8	12.0 ± 3.6	9.3 ± 5.7	21.0 ± 8.1
Heart	69.3 ± 25.2	66.5 ± 8.1	10.4 ± 5.1	37.8 ± 11.5
Lung	46.4 ± 12.6	60.8 ± 24.9	5.4 ± 3.6	23.2 ± 9.1
Blood	174.7 ± 26.1	343.7 ± 161.3	4.4 ± 3.4	22.0 ± 11.3
Muscle	114.3 ± 55.5	105.8 ± 51.7	18.6 ± 17.0	58.0 ± 26.6
Bone with marrow	25.9 ± 8.3	28.1 ± 2.7	6.9 ± 2.0	5.2 ± 0.4

### DARPin G3 tumour uptake in the presence of trastuzumab

Intravenous administration of an 18-fold molar excess of non-radiolabelled trastuzumab 24 h prior to administration of ^111^In-(HE)_3_-G3 did not alter HER2-positive tumour uptake at 4 h compared with that in control mice receiving only ^111^In-(HE)_3_-G3 (7.2 ± 1.3 vs. 7.1 ± 1.9 % ID/g, respectively; Fig. 6c). Although trastuzumab and G3 bind to domain IV of the HER2 ECD, G3 can bind in the presence of trastuzumab [23].

### MicroSPECT/CT imaging

**Fig. 7:**
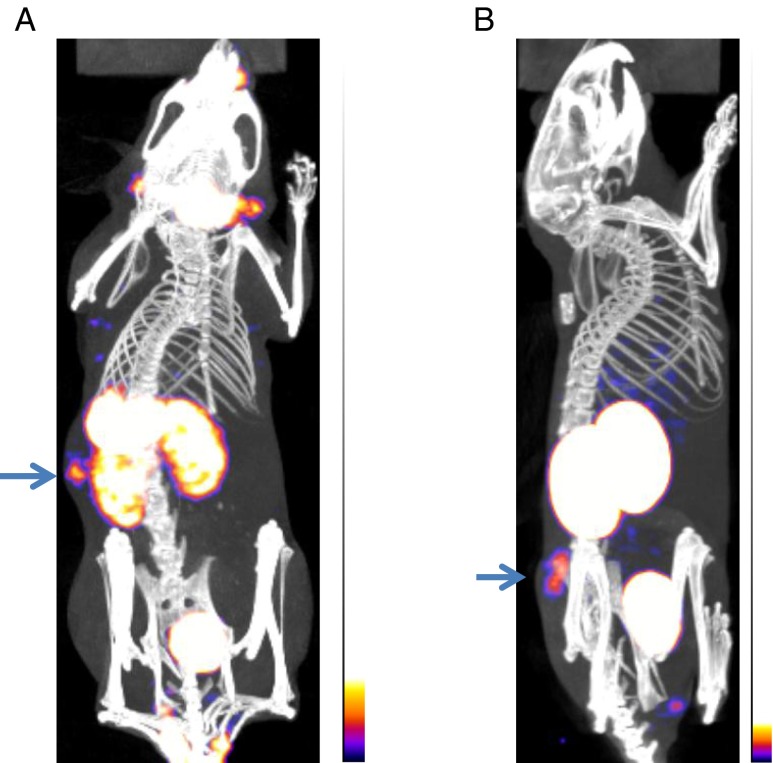
MicroSPECT/CT scans of SCID-beige mice bearing HER2-positive (BT474) tumours (*arrows*) performed at 4 h (**a** ^123^I-(HE)_3_-G3 DARPin, **b** ^111^In-(HE)_3_-G3 DARPin)

HER2 tumour uptake could be detected on the 4-h ^123^I-(HE)_3_-G3 microSPECT/CT scan but there was non-specific uptake in the stomach, kidneys, bladder and thyroid. In contrast, on the 4-h ^111^In-(HE)_3_-G3 microSPECT/CT scan, HER2 tumour uptake was detected and there was minimal non-specific uptake in non-renal organs (Fig. 7).

## Discussion

^111^In-(HE)_3_-G3 DARPin was found to be better for HER2 imaging than all other formats tested in this study. There are limitations in comparing HER2 radioligands assessed in different HER2-positive human tumour xenografts, including different levels of HER2 expression. However, within 24 h of administration to mice bearing HER2-positive tumours, ^111^In-(HE)_3_-G3 DARPin achieved higher tumour-to-blood ratios than other reported HER2 radioligands, including whole antibodies, Fab fragments of antibodies and small high-affinity proteins [28–31]. ^111^In-(HE)_3_-G3 also had greater potential for HER2 imaging than ^125^I-(HE)_3_-G3, reflected by superior microSPECT/CT scans.

The BT474 tumour-bearing mice were confirmed to have immunohistochemistry scores of 3+ with the HercepTest. The HER2-positive tumour uptake of ^111^In-(HE)_3_-G3 and ^125^I-(HE)_3_-G3 achieved at 4 h was 8.82 ± 1.25 % ID/g and 11.29 ± 3.24 % ID/g, respectively. By contrast, ^111^In-DOTA-Z_HER2:342-pep2_ achieved 39.9 percentage of injected activity per gram tissue (% IA/g) [32]. Although higher HER2-positive tumour uptake could have been anticipated, the tumour uptake was sufficient for microSPECT/CT imaging and impressive tumour-to-blood ratios were achieved.

The G3 DARPins were radioiodinated directly at their tyrosine residues. It is likely that only one of the two tyrosine residues within each G3 DARPin was radioiodinated, owing to the molar ratios of radioiodide and G3 DARPin used for radiolabelling. Although direct radioiodination is convenient and the most widely used methodology, it is recognized that the radioligand is likely to undergo in vivo dehalogenation. In future studies, we plan to compare methods using bifunctional coupling agents, to determine whether this approach can improve in vivo stability of radioiodinated G3 DARPins [33]. Nanobodies (12 – 15 kDa) are isolated from the heavy chain of Camelidae antibodies. The anti-HER2 5F7GGC Nanobody radioiodinated via the residualizing agent *N*-succinimidyl 4-guanidinomethyl 3-^125/131^I-iodobenzoate (*I-SGMIB-Nanobody) has superior targeting of HER2-positive human breast carcinoma in vitro and in vivo than its directly radioiodinated counterpart, as *I-SGMIB-Nanobody has enhanced tumour retention of radioactivity [34].

Importantly, we observed that ^111^In-(HE)_3_-G3 was able to bind to HER2 in the presence of trastuzumab. This confirms the results of structural modelling which demonstrate that DARPin G3 and trastuzumab bind to non-overlapping epitopes of HER2 domain IV [35]. Thus, ^111^In-(HE)_3_-G3 DARPin has the potential to image both treatment-naive patients and patients receiving concomitant trastuzumab without requiring a delay to treatment.

^111^In-(HE)_3_-G3 imaging would provide completely new information with the potential to improve understanding of HER2 breast cancer evolution and heterogeneity. Current literature based on biopsy sampling is inconsistent with a wide-range of reported rates of HER2 expression discordance between primary and metastatic sites, from 0 to 34 % [7]. Whole-body assessment of HER2 expression at tumour sites over time and during therapy with HER2 imaging is both more feasible and acceptable to patients than serial and multiple biopsies. Furthermore, considerably less is known about gastric cancer HER2 biology, and ^111^In-(HE)_3_-G3 imaging could help address this knowledge gap [36].

HER2 is an important therapeutic target in cancer. DARPin HER2 imaging could be used to improve the selection of patients for anti-HER2 therapy, by identifying increased HER2 expression and the need to introduce anti-HER2 therapy, as well as HER2 loss, necessitating discontinuation of anti-HER2 therapy [1]. This could not only save patients from unnecessary treatments, but also improve healthcare economics by ensuring appropriate use of expensive anti-HER2 therapies.

The pharmacokinetics of ^111^In-(HE)_3_-G3, including short half-life in serum, high tumour-to-blood ratios and low non-renal tissue uptake, are well suited to HER2 imaging. The kidneys received the highest radiation dose. However, based on medical internal radiation dose (MIRD) estimates, the maximum absorbed kidney dose in patients with the proposed clinical dose of ^111^In-(HE)_3_-G3 (300 MBq) is 1,000-fold lower than the dose associated with a 5 % rate of radiation nephritis within 5 years of administration (TD_5/5_; 9.75 mGy vs. 10 Gy) [37].

Imaging trials of most HER2 radioligands have had disappointing results due to limitations related to their pharmacokinetics for imaging which were already apparent in preclinical assessment [28–31]. It should be noted that the majority of clinical HER2 imaging studies have not assessed HER2 tumour status histologically for correlation with HER2 scans, thus limiting the evaluation of these imaging agents. Trastuzumab radiolabelled with ^64^Cu, ^89^Zr or ^111^In has demonstrated the potential for HER2 imaging by identifying known and/or previously unknown tumour lesions in patients, but lacks sensitivity compared with conventional imaging owing to a long half-life and high blood pool activity [12–14]. Fab fragments of trastuzumab were developed for HER2 imaging because their lower molecular weight is associated with a shorter half-life in serum and faster clearance of background radiation from the blood compared with trastuzumab. However, ^68^Ga-F(ab’)_2_-trastuzumab PET imaging failed to identify any tumours among four of the eight patients with metastatic HER2-positive breast cancer assessed, due to relatively high liver uptake and blood pool activity as well as potential competition with therapeutically administered trastuzumab [38].

A radiolabelled HER2 binding Affibody molecule, Z_HER2:342_ (about 7 kDa), was used to assess three patients with HER2-positive metastatic breast cancer by PET and/or SPECT imaging. Although not all known tumour lesions were identified in two patients, there was limited tumour biopsy sampling to systematically evaluate the accuracy of HER2 Affibody molecule imaging. Unfortunately, there was high background liver uptake in this initial study [39]. Recently, a phase I trial demonstrated that the second generation Affibody ^111^In-ABY-025 has improved distribution, dosimetry and accuracy in assessing patients with HER2-positive and HER2-negative metastatic breast cancer. There was a good correlation between HER2 status assessed by SPECT/CT ^111^In-ABY-025 imaging and immunohistochemistry with the HercepTest, including confirmation that a patient who had had HER2-positive primary disease developed HER2-negative metastases [15].

In our evaluation of the DARPin G3, the (HE)_3_ tag is a beneficial component as it appeared to lower normal liver uptake of the DARPin G3 without compromising uptake in other assessed normal tissues. This is advantageous for clinical application as HER2 molecular imaging requires high tumour-to-liver tissue ratios and the liver is a common site for breast cancer metastases, yet is also involved in drug metabolism and excretion. Histidine-based tags enable IMAC purification and can also be used for chelation of [^99m^Tc(CO)_3_]^+^ [21, 27]. The His_6_ tag is well established and has been used safely in patients [40], but (HE)_3_-G3 DARPin was superior to His_6_-G3. Affibody molecules with an (HE)_3_ tag also have lower normal liver uptake than counterparts with alternative histidine-based tags [24, 25]. The G3 DARPins assessed in this study only differed by the presence or composition of their tags and thus the study was able to confirm that (HE)_3_-G3 DARPin had the lowest normal liver uptake.

The mechanism of (HE)_3_ tag-mediated reduction in liver uptake of DARPin G3 has not been established. For Affibody molecules it has been proposed that positive charge and hydrophobicity in the tag play a crucial role in liver uptake [25]. However, the untagged and His_6_-tagged Affibody molecules have basic isoelectric points (pI), which are brought to a more acidic region by switching to an (HE)_3_ tag, such that the overall pI of the different Affibody constructs tested differed greatly. By contrast the (HE)_3_-tagged, His_6_-tagged and untagged G3 DARPins have similar pI values of 4.79, 5.41 and 4.71, respectively [25]. Furthermore, the (HE)_3_-tagged, His_6_-tagged and untagged G3 DARPins have similar grand average of hydropathy (GRAVY) scores of −0.12, −0.12 and +0.02, respectively, indicating that other factors are involved, while the corresponding Affibody molecules have slightly more divergent scores of −1.03, −0.97 and −0.85.

Although in preclinical studies histidine-tagged proteins have been shown to exert greater immunogenicity than their untagged counterparts, several histidine-tagged proteins have been well tolerated in human trials [40]. For example, Endostar a novel recombinant human endostatin which inhibits angiogenesis has a histidine tag, and was safe and well tolerated in large clinical trials [41]. Interestingly, a Blast search (NCBI) for a short input sequence with HEHEHE as the query, yielded a variety of hits in the database of almost identical sequences, e.g. HEHEH in the human zinc transporter (gene ID SLC39A9) and HEHEQE in kinase 3 (gene ID TAOK3). The HHHHHH sequence cannot be found using a Blast search in the *Homo sapiens* database. Instead clusters of histidine are found that are coordinated with metal ions, e.g. Ca^2+^, Mg^2+^ and Zn^2+^. The occurrence of the HEHEH sequence in human proteins could mean that the HE_3_ tag is a potentially safer alternative to the His_6_ tag.

### Conclusion

A clinically valuable radioligand for HER2 molecular imaging in breast cancer and gastric cancer would require minimal normal liver and stomach uptake, as well as the ability to bind to HER2 in the presence of concomitant anti-HER2 therapy. We have demonstrated that ^111^In-(HE)_3_-G3 DARPin has specificity for HER2, binds in the presence of trastuzumab, and achieves high tumour-to-blood ratios and reasonable tumour to non-renal tissue ratios, including tumour-to-liver and tumour-to-stomach ratios. Based on the presented preclinical data, ^111^In-(HE)_3_-G3 could realize the clinical potential of HER2 imaging and may be suitable for assessment in a first-in-human study.

## Electronic supplementary material

Below is the link to the electronic supplementary material.

ESM 1 (DOCX 357 kb)
