# Supplementary material for: Development of the designed ankyrin repeat protein (DARPin) G3 for HER2 molecular imaging
Source: Eur J Nucl Med Mol Imaging. 2014 Nov 13;42(2):288–301. doi: 10.1007/s00259-014-2940-2 (PMC4315530; doi:10.1007/s00259-014-2940-2)
Supplement: Supplementary file 1 — (DOCX 357 kb) [file 259_2014_2940_MOESM1_ESM.docx]

# Supplemental section: Development of the Designed ankyrin repeat protein (DARPin) G3 for HER2 molecular imaging

**Supplemental Materials and Methods**

**DARPin production**

DARPin G3 variants with N-terminal His_6_, (HE)_3_ or cleavable tags with a C-terminal cysteine for site-specific labelling or with a C-terminal His_6_ tag without any cysteines (G3-His_6_) were cloned and expressed in *P. pastoris* [Supplemental Fig. 1]. Briefly, a DNA fragment encoding the variant DARPin was synthesized (DNA 2.0, US), digested with *Xho*I and *Xba*I (New England Biolabs, Hitchin, Hertfordshire, UK and ligated into pPICZαB (Life Technologies, Paisley, UK) cut with the same enzymes. After transformation of *E. coli* TOP10 (Life Technologies) the plasmid was purified and linearized with *Pme*I (New England Biolabs) and transferred by electroporation into *P. pastoris* X33 cells (Life Technologies).

Fermentation was performed in a 10 L bioreactor (Bioflo 3000, New Brunswick Scientific, Eppendorf, Cambridge, UK), using basal salt medium and a glycerol feeding strategy as described elsewhere [[1](#_ENREF_1)]. The primary DARPin capture step was radial flow bed adsorption (CRIO-MD 62, Proxcys, Nieuw-Amsterdam, Netherlands) IMAC (resin, Sterogen), loaded with nickel (for cleavable tag-G3 and (HE)_3_-G3) or copper (for His_6_-G3 and G3-His_6_) [[1](#_ENREF_1), [2](#_ENREF_2)]. The captured DARPin was supplemented with 5 mM dithiothreitol (DTT), concentrated with a Labscale TFF system (Merck Millipore, Billerica, Massachusetts, US) using a Biomax 10 kDa ultrafiltration membrane (Merck Millipore) and dialysed against PBS containing 5 mM DTT (Sigma-Aldrich, St. Louis, Missouri, US). Subsequently, the monomer fraction was collected from a PBS-equilibrated 500 ml Superdex 75 column (GE Healthcare, Little Chalfont, Buckinghamshire, UK). The expressed DARPins were assessed by western blotting with the primary antibodies, mouse tetra-His antibody, BSA-free (Qiagen, Crawley, West Sussex, UK) or rabbit anti-DARPin serum (B. Dreier and A. Plückthun, unpublished). Molecular weights were confirmed by MALDI mass spectrometry. To generate the untagged G3, the His_6_ tag was cleaved from the cleavable tag-G3 by His_6_-tagged human rhinovirus (HRV) 3C protease (Merck Millipore) according to the manufacturer’s recommendations. Cleavage was confirmed by western blotting and untagged-G3 was separated from HRV 3C protease by IMAC.

**DARPin G3 DOTA-conjugation**

DARPins were conjugated with 1,4,7,10-tetraazacyclododecane-1,4,7-tris-acetic acid-10-maleimidoethylacetamide (mal-DOTA) (Macrocyclics, Dallas, Texas, US). Initially, the DARPins, still containing DTT, were treated with 1 mM EDTA in PBS for 30 min, followed by 5 mM Bond-Breaker TCEP Solution, at pH 7.4 (Thermo Fisher Scientific, Runcorn, Cheshire, UK) for 2.5 h at room temperature. To remove reducing agents, reduced DARPins were buffer-exchanged using a PD-10 size-exclusion column into 0.1 M sodium phosphate buffer, pH 7, and subsequently mixed with a 4-fold molar excess of mal-DOTA at room temperature with gentle agitation for 72 h. The resultant DOTA-conjugated DARPins were separated from excess mal-DOTA by buffer-exchange with a PD-10 size exclusion column into 0.2 M ammonium acetate buffer, pH 7, followed by centrifugation in a 2 ml Vivaspin column with a 5,000 Da MWCO polyethersulfone membrane (Sartorius Stedim, Epsom, Surrey, UK) and reconstituted in 0.2 M ammonium acetate buffer, pH 7. Finally, the conjugated DARPins were dialysed at 4 ºC against 0.2 M ammonium acetate pH 6.5 containing Chelex-100 (1% w/v) (Bio-Rad Laboratories, Hemel Hempstead, Hertfordshire, UK) using a Maxi GeBAflex-tube with a 3,500 Da MWCO membrane (Generon, Maidenhead, Berkshire, UK). The dialysis buffer was changed three times over three days. Conjugation efficiency was assessed by MALDI mass spectrometry. All solutions were treated with Chelex-100 chelating ion exchange resin prior to use.

**Supplemental Tables**

Supplemental Table 1: Stability of label in ^111^In-(HE)_3_-G3 DARPin in PBS at 4 °, 20 ° and 37 °C and in serum at 37 °C for 24 h post-radiolabelling assessed by instant thin layer chromatography.

| Time post-radiolabelling | DARPin radiolabelling (%) | Colloid (%) | Other radiolabelled products (%) |
| --- | --- | --- | --- |
| Serum stability at 37 °C |  |  |  |
| 1 h | 100 | 0 | 0 |
| 2 h | 100 | 0 | 0 |
| 4 h | 100 | 0 | 0 |
| 24 h | 100 | 0 | 0 |
| PBS stability at 37 °C |  |  |  |
| 1 h | 100 | 0 | 0 |
| 2 h | 100 | 0 | 0 |
| 4 h | 100 | 0 | 0 |
| 24 h | 96.4 | 0 | 3.6 |
| PBS stability at 20 °C |  |  |  |
| 1 h | 100 | 0 | 0 |
| 2 h | 100 | 0 | 0 |
| 4 h | 100 | 0 | 0 |
| 24 h | 97.4 | 0 | 2.6 |
| PBS stability at 4 °C |  |  |  |
| 1 h | 100 | 0 | 0 |
| 2 h | 100 | 0 | 0 |
| 4 h | 100 | 0 | 0 |
| 24 h | 95.9 | 0 | 4.1 |

Supplemental Table 2: Biodistribution of ^111^In-G3 DARPins in non–tumour bearing BALB/c mice (mean % ID/g ± SD). Four mice assessed per time point and construct. G3 DARPin with lowest uptake for each normal tissue is emboldened.

1. 4 hour post-administration of ^111^In G3 DARPins

| Tissue | Untagged-G3 ^111^In mean %ID/g at 4 h ± SD | His_6_-G3 ^111^In mean %ID/g at 4 h ± SD | (HE)_3_-G3 ^111^In mean %ID/g at 4 h ± SD |
| --- | --- | --- | --- |
| Intestine | **0.31**  **±0.11** | 0.37  ±0.07 | 0.35  ±0.06 |
| Pancreas | 0.32  ±0.01 | 0.58  ±0.10 | **0.29**  **±0.09** |
| Spleen | 1.27  ±0.37 | 0.91  ±0.08 | **0.46**  **±0.06** |
| Stomach | 0.41  ±0.14 | 0.38  ±0.27 | **0.21**  **±0.05** |
| Kidney | 305.72  ±27.25 | **235.73**  **±46.62** | 286.67  ±38.61 |
| Liver | 4.31  ±0.40 | 3.72  ±0.39 | **1.57**  **±0.04** |
| Heart | 0.34  ±0.03 | 0.42  ±0.07 | **0.27**  **±0.05** |
| Lung | 0.59  ±0.04 | 0.69  ±0.45 | **0.50**  **±0.21** |
| Blood | 0.10  ±0.02 | 0.33  ±0.24 | **0.05**  **±0.01** |
| Muscle | 0.20  ±0.07 | 0.27  ±0.09 | **0.13**  **±0.07** |
| Bone with marrow | **0.71**  **±0.33** | 0.91  ±0.14 | 0.79  ±0.39 |
| Bone marrow | 1.55  ±0.35 | 1.26  ±0.15 | **0.90**  **±0.49** |

1. 24 hour post-administration of ^111^In G3 DARPins

| Tissue | Untagged-G3 ^111^In mean %ID/g at 24 h ± SD | His_6_ -G3 ^111^In mean %ID/g at 24 h ± SD | (HE)_3_-G3 ^111^In mean %ID/g at 24 h ± SD |
| --- | --- | --- | --- |
| Intestine | 0.43  ±0.01 | 0.46  ±0.08 | **0.23**  **±0.03** |
| Pancreas | 0.26  ±0.03 | 0.45  ±0.02 | **0.24**  **±0.03** |
| Spleen | 0.99  ±0.06 | 0.87  ±0.11 | **0.38**  **±0.06** |
| Stomach | 0.32  ±0.04 | 0.47  ±0.04 | **0.11**  **±0.04** |
| Kidney | 191.95  ±11.25 | **174.19**  **±18.76** | 228.58  ±32.92 |
| Liver | 3.06  ±0.43 | 3.45  ±0.34 | **1.05**  **±0.05** |
| Heart | **0.26**  **±0.03** | 0.35  ±0.08 | 0.39  ±0.32 |
| Lung | **0.48**  **±0.09** | 0.68  ±0.56 | 0.55  ±0.27 |
| Blood | **0.05**  **±0.01** | 0.06  ±0.01 | 0.13  ±0.14 |
| Muscle | 0.22  ±0.07 | 0.17  ±0.05 | **0.16**  **±0.10** |
| Bone with marrow | 0.72  ±0.15 | **0.71**  **±0.07** | 0.79  ±0.14 |
| Bone marrow | 1.70  ±1.17 | 1.77  ±1.56 | **0.55**  **±0.10** |

Supplemental Table 3: Biodistribution of ^125^I-G3 DARPins in BALB/c mice (mean % ID/g ± SD). Four mice assessed per time point and construct. The G3 DARPin with lowest uptake for each normal tissue is emboldened.

1. 4 h post-administration of ^125^I G3 DARPins

| Tissue | Untagged-G3 ^125^I mean %ID/g ± SD at 4 h | His_6_–G3 ^125^I mean  %ID/g ± SD at 4 h | (HE)_3_ G3 ^125^I mean  %ID/g ± SD at 4 h |
| --- | --- | --- | --- |
| Intestine | **0.97**  **±0.12** | 1.76  ±0.46 | 1.05  ±0.20 |
| Pancreas | **0.65**  **±0.14** | 2.99  ±1.25 | 0.91  ±0.17 |
| Spleen | 0.81  0.09 | 1.77  ±0.62 | **0.70**  **±0.11** |
| Stomach | 18.38  ±4.47 | 22.55  ±6.81 | **10.40**  **±1.05** |
| Kidney | 4.72  ±0.40 | 6.12  ±1.12 | **2.34**  **±0.35** |
| Liver | 0.98  ±0.11 | 1.66  ±0.34 | **0.84**  **±0.35** |
| Heart | **0.53**  **±0.05** | 1.37  ±0.48 | 0.68  ±0.08 |
| Lung | **0.96**  **±0.15** | 2.26  ±0.70 | 1.45  ±0.66 |
| Blood | **1.16**  **±0.15** | 2.77  ±0.89 | 1.42  ±0.16 |
| Muscle | 0.44  ±0.27 | 1.75  ±0.72 | **0.43**  **±0.08** |
| Bone with marrow | 1.37  ±0.31 | 1.69  ±0.55 | **1.29**  **±0.40** |
| Bone marrow | **0.86**  **±0.17** | 4.03  ±1.95 | Not isolated |

1. 24 h post-administration of ^125^I G3 DARPins

| Tissue | Untagged-G3 ^125^I mean %ID/g ± SD at 24 h | His_6-_G3^125^I mean %ID/g ± SD at 24 h | (HE)_3_–G3 ^125^I mean  %ID/g ± SD at 24 h |
| --- | --- | --- | --- |
| Intestine | 0.12  ±0.04 | 0.14  ±0.06 | **0.08**  **±0.06** |
| Pancreas | 0.04  ±0.01 | 0.06  ±0.02 | **0.03**  **±0.02** |
| Spleen | 0.12  ±0.01 | 0.10  ±0.02 | **0.04**  **±0.01** |
| Stomach | **0.26**  **±0.10** | 0.50  ±0.29 | **0.26**  **±0.18** |
| Kidney | 1.02  ±0.09 | 1.26  ±0.17 | **0.19**  **±0.03** |
| Liver | 0.21  ±0.04 | 0.23  ±0.04 | **0.01**  **±0.03** |
| Heart | **0.04**  **±0.02** | 0.06  ±0.02 | **0.04**  **±0.01** |
| Lung | 0.09  ±0.02 | 0.11  ±0.02 | **0.07**  **±0.02** |
| Blood | 0.08  ±0.03 | 0.10  ±0.04 | **0.07**  **±0.04** |
| Muscle | **0.02**  **±0.001** | 0.03  ±0.01 | **0.02**  **±0.01** |
| Bone with marrow | 0.46  ±0.11 | **0.42**  **±0.11** | 0.85  ±0.28 |
| Bone marrow | Weight too low | **0.30**  **±0.20** | Not isolated |

Supplemental Table 4: Biodistribution of ^125^I-(HE)_3_ G3 DARPin and ^111^In-(HE)_3_ G3 DARPin in tumour bearing mice (mean % ID/g ± SD). Four mice assessed per time point and construct.

| Tissues | ^125^I-(HE)_3_-G3 mean %ID/g ± SD at 4 h | ^111^In-(HE)_3_-G3 mean %ID/g ± SD at 4 h | ^125^I-(HE)_3_-G3 mean %ID/g ± SD at 24 h | ^111^In-(HE)_3_ G3 mean %ID/g ± SD at 24 h |
| --- | --- | --- | --- | --- |
| Intestine | 1.65  ±0.81 | 0.30  ±0.20 | 0.13  ±0.04 | 0.40  ±0.15 |
| Pancreas | 2.34  ±1.78 | 0.18  ±0.03 | 0.07  ±0.02 | 0.21  ±0.08 |
| Spleen | 1.76  ±1.19 | 0.28  ±0.05 | 0.07  ±0.01 | 0.29  ±0.07 |
| Stomach | 12.76  ±11.91 | 0.08  ±0.04 | 0.45  ±0.25 | 0.16  ±0.08 |
| Kidney | 3.18  ±1.78 | 232.02  ±24.11 | 0.25  ±0.01 | 196.55  ±31.02 |
| Liver | 1.55  ±0.96 | 0.72  ±0.11 | 0.12  ±0.02 | 0.72  ±0.23 |
| Heart | 1.29  ±0.70 | 0.14  ±0.03 | 0.07  ±0.01 | 0.12  ±0.03 |
| Lung | 2.77  ±1.75 | 0.20  ±0.04 | 0.11  ±0.04 | 0.15  ±0.06 |
| Blood | 3.55  ±2.20 | 0.05  ±0.01 | 0.13  ±0.04 | 0.03  ±0.02 |
| Muscle | 0.99  ±0.67 | 0.09  ±0.04 | 0.05  ±0.02 | 0.09  ±0.05 |
| Bone with marrow | 1.76  ±0.80 | 0.36  ±0.11 | 0.47  ±0.12 | 0.27  ±0.02 |
| Tumour | 11.29  ±3.24 | 8.82  ±1.25 | 2.45  ±0.60 | 8.07  ±0.93 |

**Supplemental Figures**

Supplemental Fig. 1: DARPin constructs used in the present study. All were prepared from *P. pastoris* as a fusion protein downstream of the pre-pro sequence of *S. cerevisiae* alpha factor, which ends in ...QKR (not shown in sequence) and is cleaved off to result in the final secreted protein sequences shown. Pre-cleavage denotes the fusion where the pre-pro-sequence is fused to the spacer EAEA, (partially) cleaved off by *Pichia* DPAPase A, a His_6_ tag for purification, a Gly_3_ spacer, and the recognition site for rhinovirus 3C protease. Untagged-G3 DARPin denotes the final product after cleavage with 3C protease. In His_6_-G3 DARPin and (HE)_3_-DARPin, the respective tags are directly fused to the DARPin and not cleaved. Note that all constructs have a C-terminal gly-gly-cys tag for maleimide labelling. The two Tyr that can be labelled with iodine are highlighted (grey), the first and last residues of the DARPin G3 are emboldened and the common residues are underlined.

| **Pre-cleavage** | EAEAHHHHHHGGGSGLEVLFQGPGS**D**LGKKLLEAARAGQDDEVRILMANGAD |
| --- | --- |
| **Untagged G3** | GPGS**D**LGKKLLEAARAGQDDEVRILMANGAD |
| **His_6_-G3** | HHHHHHGPGS**D**LGKKLLEAARAGQDDEVRILMANGAD |
| **(HE)_3_-G3** | HEHEHEGPGS**D**LGKKLLEAARAGQDDEVRILMANGAD |
|  |  |
| **Pre-cleavage** | VNAKDEYGLTPLYLATAHGHLEIVEVLLKNGADVNAVDAIGFTPLHLAAF |
| **Untagged G3** | VNAKDEYGLTPLYLATAHGHLEIVEVLLKNGADVNAVDAIGFTPLHLAAF |
| **His_6_-G3** | VNAKDEYGLTPLYLATAHGHLEIVEVLLKNGADVNAVDAIGFTPLHLAAF |
| **(HE)_3_-G3** | VNAKDEYGLTPLYLATAHGHLEIVEVLLKNGADVNAVDAIGFTPLHLAAF |
|  |  |
| **Pre-cleavage** | IGHLEIAEVLLKHGADVNAQDKFGKTAFDISIGNGNEDLAEIL**Q**KLNGGC |
| **Untagged G3** | IGHLEIAEVLLKHGADVNAQDKFGKTAFDISIGNGNEDLAEIL**Q**KLNGGC |
| **His_6_-G3** | IGHLEIAEVLLKHGADVNAQDKFGKTAFDISIGNGNEDLAEIL**Q**KLNGGC |
| **(HE)_3_-G3** | IGHLEIAEVLLKHGADVNAQDKFGKTAFDISIGNGNEDLAEIL**Q**KLNGGC |

Supplemental Fig. 2: Autoradiography of SDS-PAGE gel of ^111^In-(HE)_3_-G3 stored in PBS at 4 °, 20 ° and 37 °C and human serum at 37 °C at 24 h post-radiolabelling. The serum sample was prepared with equal proportions of human serum and ^111^In-(HE)_3_-G3 in PBS, while PBS samples were non-diluted (MW: molecular weight marker).


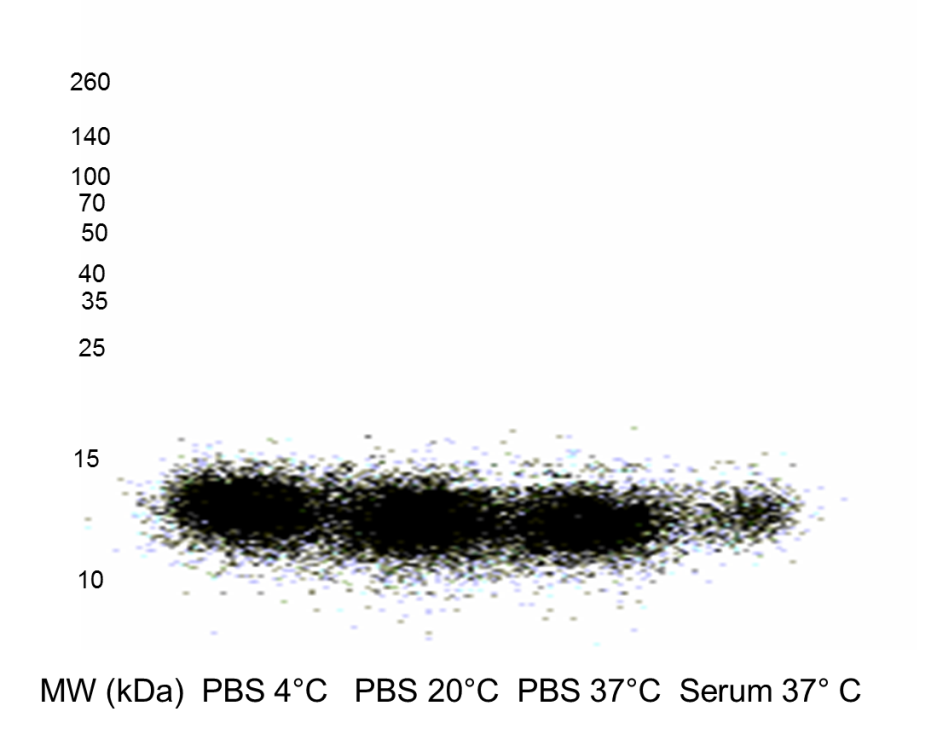


Supplemental Fig. 3: Biodistribution of ^111^In-(HE)_3_-G3 in female BALB/c mice and female SCID-beige mice bearing HER2-positive human breast tumours (BT474) at 4 and 24 h post-administration. Four mice assessed per construct at each time point. Data are presented as mean % ID/g ± SD.


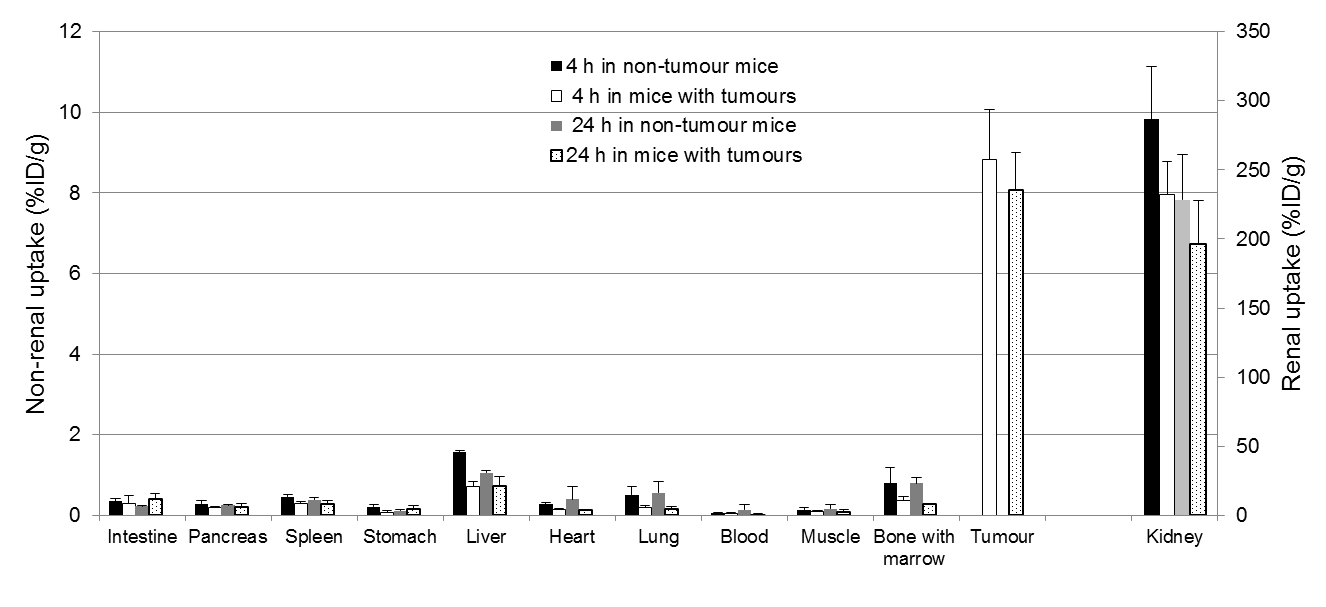


Supplemental Fig. 4: Biodistribution of ^125^I-(HE)_3_-G3 in female BALB/c mice and female SCID-beige mice bearing HER2-positive human breast tumours (BT474) at 4 and 24 h post-administration. Four mice assessed per construct at each time point. Data are presented as mean % ID/g ± SD.


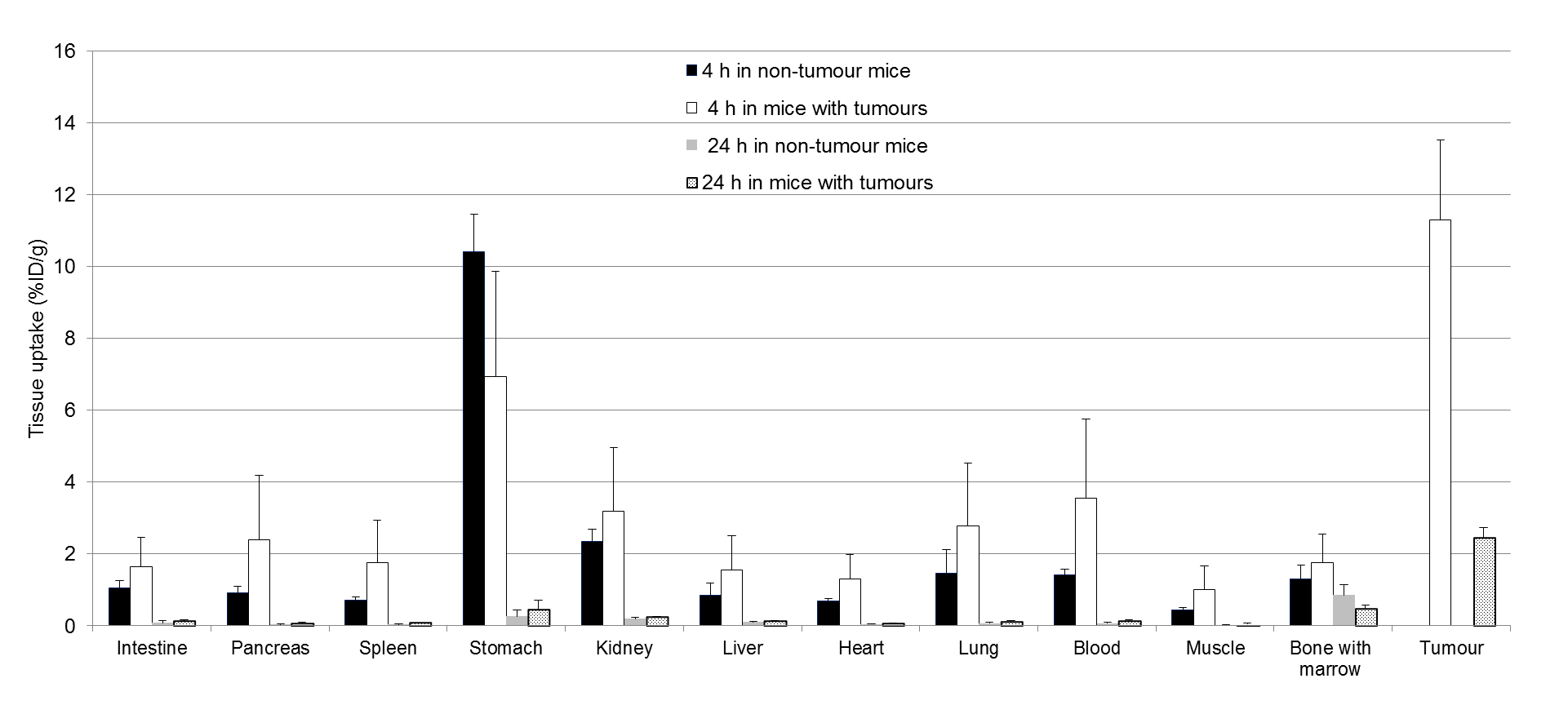


# Supplemental References

1. Tolner B, Smith L, Begent RH, Chester KA. Production of recombinant protein in Pichia pastoris by fermentation. Nature protocols. 2006;1:1006-21. doi:10.1038/nprot.2006.126.

2. Tolner B BG, Foster B, Vigor K and Chester KA Laboratory Protocols in Fungal Biology: Current Methods in Fungal Biology. New York: Springer Science & Business Media 2012.
